# Acupuncture for the Management of Chemotherapy-Induced Peripheral Neuropathy

**Published:** 2012-05-01

**Authors:** Constance Visovsky

**Affiliations:** From University of South Florida, College of Nursing, Tampa, Florida

Review of "Evaluation of acupuncture in the management of chemotherapy-induced peripheral neuropathy," by G. K. Donald, I. Tobin, and J. Stringer (2011). Acupuncture in Medicine, 29, 230–233. For a discussion of the potential threats to validity that exist in studies using a retrospective design, see the related article by Cindy Tofthagen, PhD, ARNP, AOCNP®, on page 181.

Peripheral neuropathy has been defined as a dysfunction of the sensory, motor, or autonomic nerves of the peripheral nervous system (Postma & Heimans, 2000). Chemotherapy-induced peripheral neuropathy (CIPN) continues to present a significant challenge to patients and health-care providers. The incidence of CIPN is difficult to estimate due to the lack of gold-standard definitions and measures. However, it is estimated that severe CIPN occurs in 3% to 7% of patients treated with single agents and upward of 38% of those treated with multiple chemotherapeutic agents (Cavaletti & Zanna, 2002).

Patients with CIPN report such symptoms as paresthesia and dysesthesia, cold sensitivity, pain, loss of deep tendon reflexes, impairment of gait and balance, difficulty with fine motor skills, urinary and sexual dysfunction, and alterations in orthostatic blood pressure (Stubblefield et al., 2009).

Despite many clinical trials testing various agents for the prevention and treatment of CIPN, effective pharmacologic therapies are lacking; none tested can be recommended for adoption into clinical practice (Visovsky, Collins, Abbott, Ashenbrenner, & Hart, 2007). Clinical trials of agents for CIPN are plagued by a lack of standardized reporting of CIPN, flaws in study design, small sample sizes, and varied chemotherapeutic agents administered (Stubblefield et al., 2009). Thus, determining an effective pharmacologic therapy to ameliorate CIPN requires further investigation. At the same time, studies examining the effectiveness of nonpharmacologic treatments for CIPN are being explored.

The mechanism by which acupuncture serves to relieve the symptoms of peripheral neuropathy remains unclear. However, acupuncture has been linked to substances that are associated with nervous system activity, such as beta-endorphin, gamma-aminobutyric acid, glutamate, adenosine, and nerve growth factor (Zhao, 2008; Goldman et al., 2010). Zhao (2008) also demonstrated that acupuncture activates both myelinated and unmyelinated nerve fibers. Studies on the use of acupuncture to combat symptoms related to peripheral neuropathy have been conducted in patients with diabetes (Ahn, Bennani, Freeman, Hamdy, & Kaptchuk, 2007; Zhang, Ma, & Yan, 2010; Tong, Guo, & Han, 2010).

## Acupuncture for CIPN

At an acute care hospital in England, Donald, Tobin, & Stringer (2011) explored the clinical effectiveness of acupuncture in the treatment of CIPN refractory to standard care as a component of an evaluation of the acupuncture service given to patients as part of routine hospital care. Eighteen patients with CIPN, who were referred by physicians or nurse specialists or self-referred, were offered a six-session course of acupuncture—a standard service provided at the hospital facility. To ensure safety, patients were assessed for the ability to tolerate acupuncture (i.e., platelet count > 100 × 10^9^/L). The treatments were applied by trained nurses who applied the acupuncture needles to specific points based on individual patient presentation. Once inserted, the acupuncture needles were then left in place for 30 to 45 minutes

The acupuncturist performed an evaluation of each patient’s CIPN symptoms at the first session. The final evaluation, performed after the sixth session, was carried out by a different member of the acupuncture team to minimize bias. Treatment response to acupuncture, gathered by patient report, was categorized as improved, unchanged, aggravated, increased, or other. Additional benefits reported by patients were collected using an itemized checklist (relaxation, better sleep, reduced stress, improved mood, less medication, and other). As the primary purpose was to evaluate the acupuncture service offered to patients, there were no standard questionnaires used, and there was no attempt to statistically control for variables that could influence results. Following the course of treatment, the patient’s electronic medical record was accessed for demographic data and the chemotherapeutic agent most likely responsible for neuropathy. Demographic and medical data of the patient population examined are reported in Table 1.

**Table 1:**
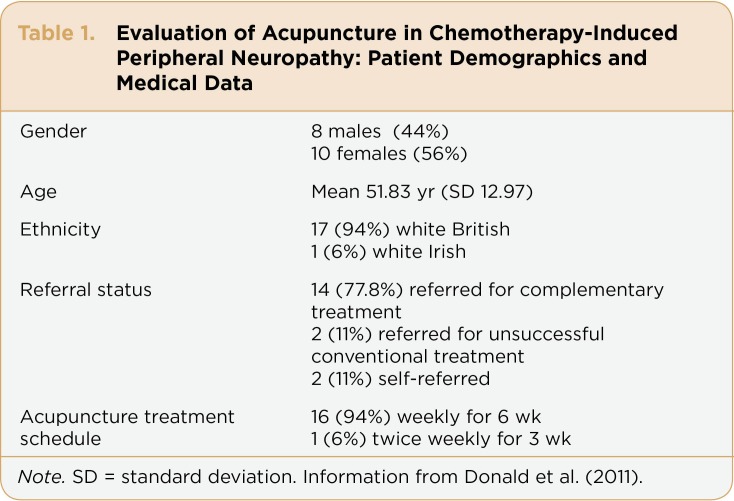
Table 1. Evaluation of Acupuncture in Chemotherapy-Induced Peripheral Neuropathy: Patient Demographics and Medical Data

## Results

Seventeen patients completed all six acupuncture sessions. One patient died during the six-treatment interval. Of those who completed all sessions, 14 patients (82%) reported that the acupuncture treatment improved CIPN symptoms, whereas 3 patients (18%) reported no change in their CIPN symptoms. There were no reports of aggravated or increased CIPN symptoms. Six (35%) patients reported receiving one additional benefit from acupuncture, seven patients (41%) reported receiving more than one additional benefit, and four patients (24%) noted receiving no additional benefits. The additional benefits reported were better sleep (47%), relaxation (47%), reduced stress (24%), less medication (24%), improved mood (6%), and other benefits (24%). A variety of acupoints were used by the acupuncture team in the treatment of CIPN. The most common acupoints utilized were SP6 and ST36, which were used in all patients, and LV3, which was used in 14 of 18 patients.

Of the patients receiving oxaliplatin, 100% (n = 4) reported improvement in oxaliplatin-induced peripheral neuropathy following the 6-week acupuncture treatment. Sixty percent (3 of 5 patients) of patients receiving vincristine reported improvement in CIPN symptoms, whereas 75% (3 of 4 patients) of patients receiving bortezomib (Velcade) reported improvement. Of the two patients receiving thalidomide (Thalomid), 100% reported relief of CIPN symptoms. Overall, 86% of patients with myeloma reported improvement in symptomatic CIPN regardless of the chemotherapeutic agent received.

## Clinical Implications

Translating reports of research and clinical investigations into practice can be difficult. This is especially true in investigations of treatments for CIPN, due to methodologic issues associated with past standard clinical trials and scant data to support the application of nonpharmacologic therapies.

In exploring the use of acupuncture to treat CIPN, only one case series and one case report could be found. Five patients with advanced gynecologic cancer treated with carboplatin and paclitaxel were given acupuncture treatment for symptomatic CIPN once weekly for 6 weeks, followed by 4 weeks of rest, and a second course of acupuncture for 6 additional weeks. Following acupuncture treatment, gait was significantly improved in three patients with balance disturbance. Improvements in sensation and decreased analgesic use were demonstrated all five patients involved. Control of symptoms, including pain, numbness, and tingling of fingers and toes, was obtained after only one treatment. There were no adverse side effects, and the benefits of acupuncture were maintained for 6 months for four of the five patients involved (Wong & Sagar, 2006).

In a case report of a 48-year-old man with multiple myeloma and bortezomib-induced peripheral neuropathy, acupuncture delivered once weekly for 6 weeks was successful in relieving painful neuropathy (Bao, Zhang, Badros, & Lao, 2011).

## Conclusions

At this time, there are insufficient data and controlled clinical trials to recommend acupuncture for the treatment of CIPN. However, because there are no evidence-based pharmacologic agents that can be recommended, and as previous studies in patients with diabetes and cancer demonstrated no adverse events from acupuncture, further studies of this option should be explored. Well-designed, randomized trials are needed to further demonstrate the effectiveness of this potentially promising intervention.
